# Supplementary material for: Cost-effectiveness analysis of an intervention project engaging Traditional and Religious Leaders to improve uptake of childhood immunization in southern Nigeria
Source: PLoS One. 2021 Sep 16;16(9):e0257277. doi: 10.1371/journal.pone.0257277 (PMC8445457; doi:10.1371/journal.pone.0257277)
Supplement: Attachment — Submitted filename: RESPONSE TO REVIEWERS - CEA.docx [file pone.0257277.s001.docx]

RESPONSE TO REVIEWERS

Reviewer #1: The study objective is to examine the effectiveness and cost-effectiveness of using a multi-faceted intervention involving traditional and religious leaders for community engagement to improve uptake of routine immunisation services in Southern Nigeria.

As a cluster-randomised controlled clinical trial, there are a number of major methodological issues:

1. The authors had not elaborated the calculation and justification of the sample size estimation basing on a suitable anticipated effect size for the cluster randomization design.

RESPONSE: Reference has been made to the already published effectiveness study.

2. Although LGAs and wards were said to be randomly selected, the process of randomization has not been clearly explained.

RESPONSE: Reference has been made to the already published the effectiveness study

3. How was the outcome of vaccine uptake rate being ascertained in the participating villages? Were those routinely collected data, or collected and ascertainment with a specific procedure in this study? How was the variability of the completeness and accuracy of the data of ascertainment in different villages? In particular, would there be a differential degree of outcome ascertainment between intervention and control villages?

RESPONSE: Reference the effectiveness study.

4. Many of the outcomes were not clearly defined and elaborated on how they were ascertained. This included how “timely vaccination” was defined, and how was “Number of Measles cases averted” and “Number of children who could benefit” be ascertained?

RESPONSE: The definition of timely vaccination allowed for no more than two weeks after the scheduled vaccination date.

The number of measles cases averted was estimated based on the number of cases of measles reported from the survey.

Number of children who could benefit was the final number of children recruited in the end line sample in all of the intervention wards as seen in the effectiveness study.

5. Was the study method, especially the presence of a control group being know by all participating villages? Would there be any bias on the observed outcome in either direction as a result of the awareness?

RESPONSE: Reference the effectiveness study. Non-contiguous villages were selected. It is difficult to say if there would be any bias such awareness would have created.

6. How much may the better vaccination uptake be attributed to a potential Hawthorne effect?

RESPONSE:

It is highly unlikely that the better vaccination uptake observed can be attributed to a potential Hawthorne as survey respondents and outcome assessors were blinded to intervention assignment.

7. The method of statistical analysis of data has not been explained.

RESPONSE: The effect of the intervention was estimated using logistic regression including random effects to adjust for non-independence of clustered observations at village, ward and LGA level.

8. The calculation of the counterfactual effect as reported on Table 2 has not been clearly explained. Is that just a simple subtraction of the effect between the intervention and control group? The figures does not exactly adds up.

RESPONSE: Patrick please help: Yes, the counterfactual was calculated as suggested. The small discrepancies arose because of rounding of data points.

9. Can the authors also discuss on the extent of external validity of the result, including both the nature of the (or a similar) intervention, and the observed clinical effectiveness and cost-effectiveness of the intervention, to other villages and areas of Nigeria or countries with a comparable degree of socio-economic development, or to other more developed areas/ countries.

RESPONSE: The external validity has been commented on in the last paragraph of the discussion. It reads as follows: “Similar settings (like what is obtainable in most low-and middle-income countries) may have similar impact of the intervention. The intervention is more likely to be impactful where the traditional and religious leadership are embedded in one system. Furthermore, even though the TRL in Cross River State are under the same functional relationship, there are large differences between TRL in different wards. So, we could expect to see similar results in areas that have different TRL setups. However, it is difficult to ascertain how effective each component of the intervention would be and the cost-effectiveness of implementing single components of the intervention.”

Reviewer #2: The authors have carried out an economic evaluation of a community-based interventions engaging traditional and religious leaders to improve immunization uptake in a state of Nigeria. it is good that the cost data was collated during the execution of a cluster randomized trial.
The following lines were unclear or could do with some explanation:

1. Line 101 point 2 on community engagement does not make sense: "Routine community for a were used by the TRLs to share information on routine immunization "

RESPONSE: Corrected to read: “Routine community engagements were used by the TRLs to share information on routine immunization as a means of encouraging community members to get their children vaccinated.”

2. It would be useful if the authors could expand on lines 170-172 on how they estimated cost of measles illness "The estimate for averting measles was estimated based on the estimated short-term cost of measles illness (i.e. cost of treatment, transport, caretaker lost wages). This was estimated to be7 US$ per care-seeking case averted"

RESPONSE:

This estimate was derived from a previous study. This has been rephrased to explain how it was derived in the sentence. Thus it now reads

“The estimate cost for averting measles illness was obtained from an earlier study based on the forecasted short-term cost that would be incurred for treatment of measles illness (i.e. cost of treatment, transport, caretaker lost wages) divided by the number of individuals with measles disease who likely sought health-care treatment. In the study the different parameters was derived from country-specific estimates of proportions of children for whom care was sought for measles as well as data on the duration and rates of hospital admission while the estimated cost were primarily obtained from the WHO’s Choosing Interventions that are Cost-Effective (WHO-CHOICE) project for the countries. This was estimated to be 7 US$ per care-seeking case averted”

3. Likewise it would be useful if they elaborated on how they estimate number of measles cases averted? (lines 172-74) "estimated based on the number of cases of measles reported on the District Health Information System."

RESPONSE: The number of measles averted was the final counterfactual estimate from the end line survey and not from the District Health Information System. This has been corrected in the manuscript.

4. Re: line 245-48: “The findings showed that the total cost of the intervention for the eight wards where this was carried out was US$15,189 giving a unit cost of US$12 for every child covered within the wards”. Was there variation in unit costs between different wards? While the aggregate number is useful for deriving a unit cost, it would be interesting to know if unit cost varied between wards and if so how much?

RESPONSE: costing was not done per ward but per Local Government. There was no variation in the cost between the LGAs.

5. Line 253: "Similarly, it revealed that to have one more child to be vaccinated with all the vaccine antigens including measles antigen on time in the intervention group compared to the control group US$102 will need to be spent." it would be useful if the authors explained this.

RESPONSE: It has been rephrased and written as “Similarly, it revealed that it will cost US$102 to have one more child fully vaccinated in the intervention group. With this amount there will be a reduction by one of the number of children who have not been fully immunized.”

6. Line 283: "Thus it is recommended, especially in low and middle income countries that have a higher level of informal governance structures to consider the use of this strategy in improving their immunization coverage especially in hard to reach and rural areas where such structures operate maximally and which tend to lag in immunization coverage compared to urban areas."
   The evidence pertains to the context of Nigeria but is it appropriate to make a sweeping generalization to LMICs? Considering the study was carried out in one of 36 states of Nigeria.

RESPONSE: Statement revised to read “Thus it is recommended, that countries with a higher level of informal governance structures to consider the use of this strategy in improving their immunization coverage especially in hard to reach and rural areas where such structures operate maximally and which tend to lag in immunization coverage compared to urban areas.
